# Discovery of the Postcoital Contraceptive Effect of *Rhus pentaphylla* in a Study of Acute, Subchronic, and Neurodevelopmental Toxicity of Ethanolic Leaf Extract

**DOI:** 10.1002/cbdv.202501214

**Published:** 2025-08-28

**Authors:** Fatimazahra Agouram, Aicha Ezoubeiri, Abderrahman Chait, Stefania Garzoli, Zahra Sokar

**Affiliations:** ^1^ Laboratory of Pharmacology, Neurobiology, Anthropology and Environment, Semlalia Faculty of Sciences Cadi Ayyad University Marrakech Morocco; ^2^ Laboratory of Medical Analyzes Hospital Ibn Tofail Marrakech Morocco; ^3^ Department of Chemistry and Technologies of Drug Sapienza University Rome Italy

**Keywords:** acute toxicity, contraceptive, HPLC, neurodevelopmental toxicity, postcoital, *Rhus pentaphylla*, sub‐chronic toxicity

## Abstract

In the present study, the acute, subchronic, and neurodevelopmental toxicity of *Rhus pentaphylla*, a Moroccan medicinal plant, was investigated in Swiss albino mice. HPLC analysis showed the presence of phenolic compounds with rutin as the principal component. The ethanolic leaf extract showed low acute toxicity with a lethal dose 50 (LD_50_) of more than 5 g/kg. In subchronic toxicity tests, the extract resulted in an increase in body weight without affecting the biochemical parameters or tissue structure and architecture of the liver and kidneys. In addition, complete inhibition of postcoital pregnancy was observed at doses of 300 mg/kg and above. Finally, neurodevelopmental toxicity assessment revealed improved offspring body weight and behavioral performance. These results suggest that *R. pentaphylla* has promising potential as a natural fertility control product due to its significant postcoital contraceptive effects and favorable toxicity profile.

## Graphical abstract

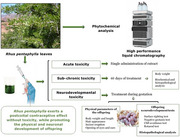

## 1 Introduction

The use of medicinal plants is widespread, particularly in developing countries, where they are perceived as natural and less toxic [1, 2]. However, their safety is not always guaranteed, especially during pregnancy, a period highly sensitive to the potential effects of phytocompounds [3]. Several studies have highlighted harmful effects of certain plants on reproduction and fetal development [4, 5], such as fenugreek *Trigonella foenum‐graecum*, which is known for its teratogenic and abortifacient properties [6, 7], as well as *Momordica charantia*, *Origanum vulgare* [8, 9], and other plants known to cause malformations and miscarriages [10, 11]. Other plants, including *Moringa oleifera*, *Persea americana*, and *Centella asiatica*, have shown neuroprotective potential promoting early neurological development [12, 13, 14, 15]. These findings emphasize the need for rigorous evaluation of medicinal plants' effects on pregnancy and neurodevelopment.

*Rhus pentaphylla*, a species belonging to the Anacardiaceae family, is commonly used in both food and traditional medicine. Locally known as tizgha or sumac in some regions of Morocco, it typically grows in non‐agricultural areas and is found across several Mediterranean countries, including Morocco, Algeria, Tunisia, and Spain [16, 17, 18]. *R. pentaphylla* is rich in flavonoids, tannins, and coumarins, and exhibits various pharmacological properties such as anti‐inflammatory, antimicrobial, antifungal, antimalarial [19], and antidiarrheal activities [20]. Traditionally, it is used in the form of decoctions prepared from its roots, leaves, bark, and fruits, primarily to relieve gastric and gastrointestinal disorders [21].

Due to its beneficial effects on digestion, *R. pentaphylla* is sometimes used by pregnant women to alleviate digestive disorders related to hormonal imbalances. However, preliminary observations from our laboratory suggested that it may interfere with reproductive processes. Indeed, inhibition of pregnancy development postmating was observed in mice treated with a leaf extract of *R. pentaphylla*, indicating a potential postcoital contraceptive effect. This use raises concerns regarding the safety of the plant during physiologically sensitive periods such as pregnancy. To date, no comprehensive toxicological studies have been conducted, particularly in pregnant females, making rigorous scientific evaluation essential to ensure safe usage.

Interestingly, alongside this inhibitory effect on gestation, the *R. pentaphylla* extract was also associated with improved physical and behavioral development of the offspring. This dual activity, both contraceptive and neurodevelopmental, suggests the involvement of endocrine or neurohormonal mechanisms that may influence both embryonic implantation and maturation of the central nervous system. The presence of bioactive compounds in the plant could thus play a role in simultaneously modulating fertility and brain development, opening new perspectives on the pleiotropic effects of traditionally used medicinal plants.

In this context, the search for plant‐based contraceptives is of major strategic importance, not only to diversify family planning options but also to meet the growing demand for natural solutions perceived as safer and culturally acceptable. Despite increasing interest, scientific data on the efficacy and safety of contraceptive plants remain limited. This study aims to fill this gap by investigating the postcoital effects of *R. pentaphylla* extract while assessing its impact on offspring development, with the goal of identifying molecules with dual action: fertility regulation and promotion of neurological development.

The primary objective of this study was to assess the safety of the ethanolic leaf extract of *R. pentaphylla* in Swiss albino mice through acute, subchronic, and neurodevelopmental toxicological analyses, in a context where no scientific data had previously guaranteed its innocuity. However, during experimentation, an unexpected inhibition of pregnancy development was observed, leading to a secondary objective: to investigate the extract's postcoital contraceptive effect. Through this integrated approach, our work aims to provide rigorous data on the safety and potential effects of *R. pentaphylla* on both fertility and the neurobehavioral development of offspring, thus clarifying its traditional use and identifying potential bioactive compounds with dual action.

## 2 Results and Discussion

### 2.1 High‐Performance Liquid Chromatography Analysis

**FIGURE 1:**
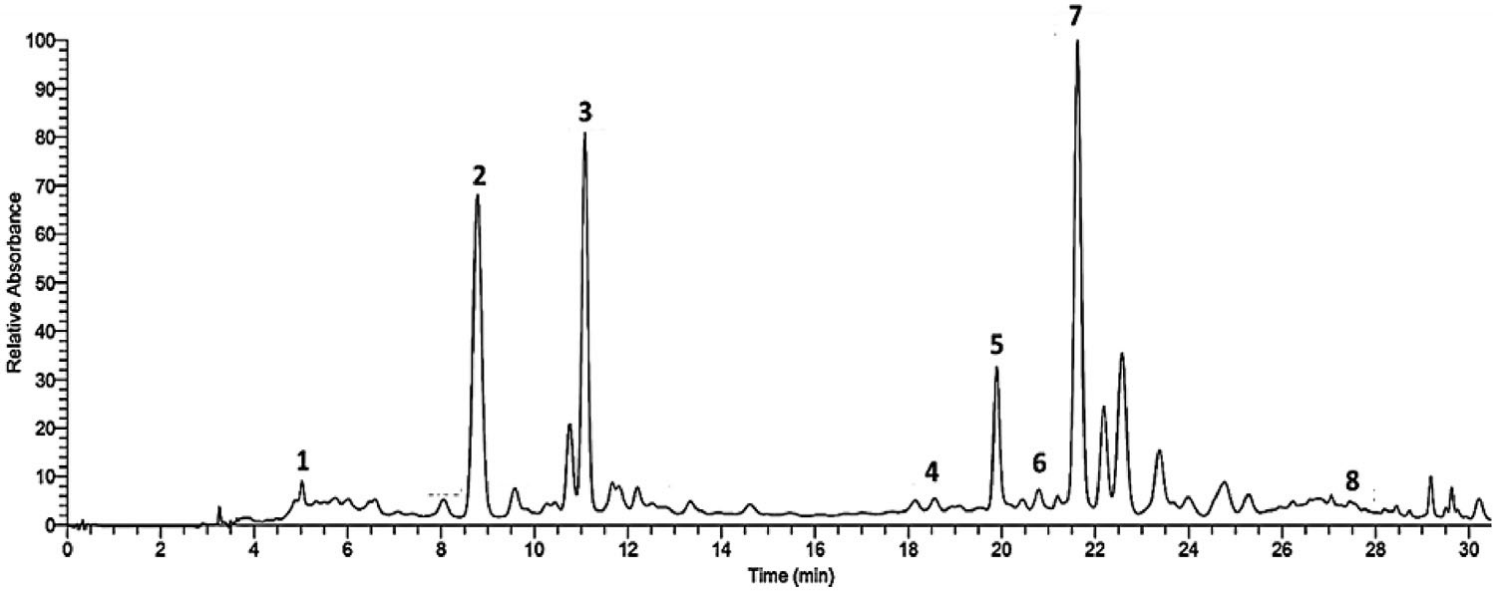
HPLC chromatogram of ethanolic extract of *Rhus pentaphylla* leaf. (1) Gallic acid; (2) epicathechin; (3) caffeic acid; (4) *p*‐coumaric acid; (5) ferulic acid; (6) hesperidin; (7) rutin; (8) kaempferol.

High‐performance liquid chromatography (HPLC) analysis identified the polyphenols in the ethanolic extract of *R. pentaphylla* leaves based on the retention times of standard compounds. The polyphenols detected in this extract include gallic acid, epicatechin, caffeic acid, *p*‐coumaric acid, ferulic acid, hesperidin, rutin, and kaempferol (Figure 1).

### 2.2 Acute Toxicity

The acute toxicity study showed that *R. pentaphylla* leaves caused no clinical signs of toxicity in any of the animals, and no mortality was observed during the 14‐day monitoring period. Therefore, the LD_50_ of *R. pentaphylla* leaves is greater than 5 g/kg when administered orally.

### 2.3 Subchronic Toxicity

#### 2.3.1 Assessment of Body Weight and Biochemical Parameters

**FIGURE 2:**
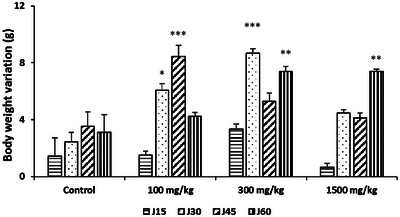
Effect of *Rhus pentaphylla* leaf on the body weight variation in mice treated for 60 consecutive days. The data are shown as mean ± SEM. ***p < 0.001, **p < 0.01, *p < 0.05 versus control.

To investigate the subchronic toxicity effect of *R. pentaphylla* treatment, we analyzed various physical and biochemical parameters. Chronic oral administration of *R. pentaphylla* leaves to mice at doses of 100, 300, or 1500 mg/kg resulted in a significant increase in body weight on Days 30 and 45 in the group treated with 100 mg/kg. However, no increase was observed in this group on Day 60. At a dose of 300 mg/kg, a significant increase in body weight was observed on Days 30 and 60 compared to the control group, while a dose of 1500 mg/kg caused a significant increase in body weight on Day 60. The weight gains thus varied according to the administered doses. No significant differences were observed in the biochemical parameters of the mice (Figure 2; Table 1). Macroscopic examination of the organs revealed no lesions or changes in appearance or color.

**TABLE 1:** Biochemical parameters of the mice.

Biochemical parameters	Groups			
	Control	100 mg/kg	300 mg/kg	1500 mg/kg
Glycemie	2.75 ± 1.13	1.44 ± 0.04	1.56 ± 0.15	1.78 ± 0.07
Urea	0.70 ± 0.14	0.69 ± 0.05	0.59 ± 0,03	0.76 ± 0.01
Creatinine (mg/L)	1.66 ± 0.66	1.33 ± 0.33	1.32 ± 0.04	1.00 ± 0.00
ALT (UI/L)	72.66 ± 2.33	71.33 ± 1.45	44.00 ± 3.78	36.00 ± 2.00
AST (UI/L)	303.66 ± 19.07	309.66 ± 20.86	254.00 ± 13.00	246.50 ± 16.50
ALP (UI/L)	107.33 ± 18.11	89.66 ± 12.11	106.66 ± 11.31	164.00 ± 15.00

*Note*: The data are presented as mean ± SEM.

Abbreviations: ALP, alkaline phosphatase; ALT, alanine transaminase; AST, aspartate aminotransferase.

### 2.4 Histopathological Examination

**FIGURE 3:**
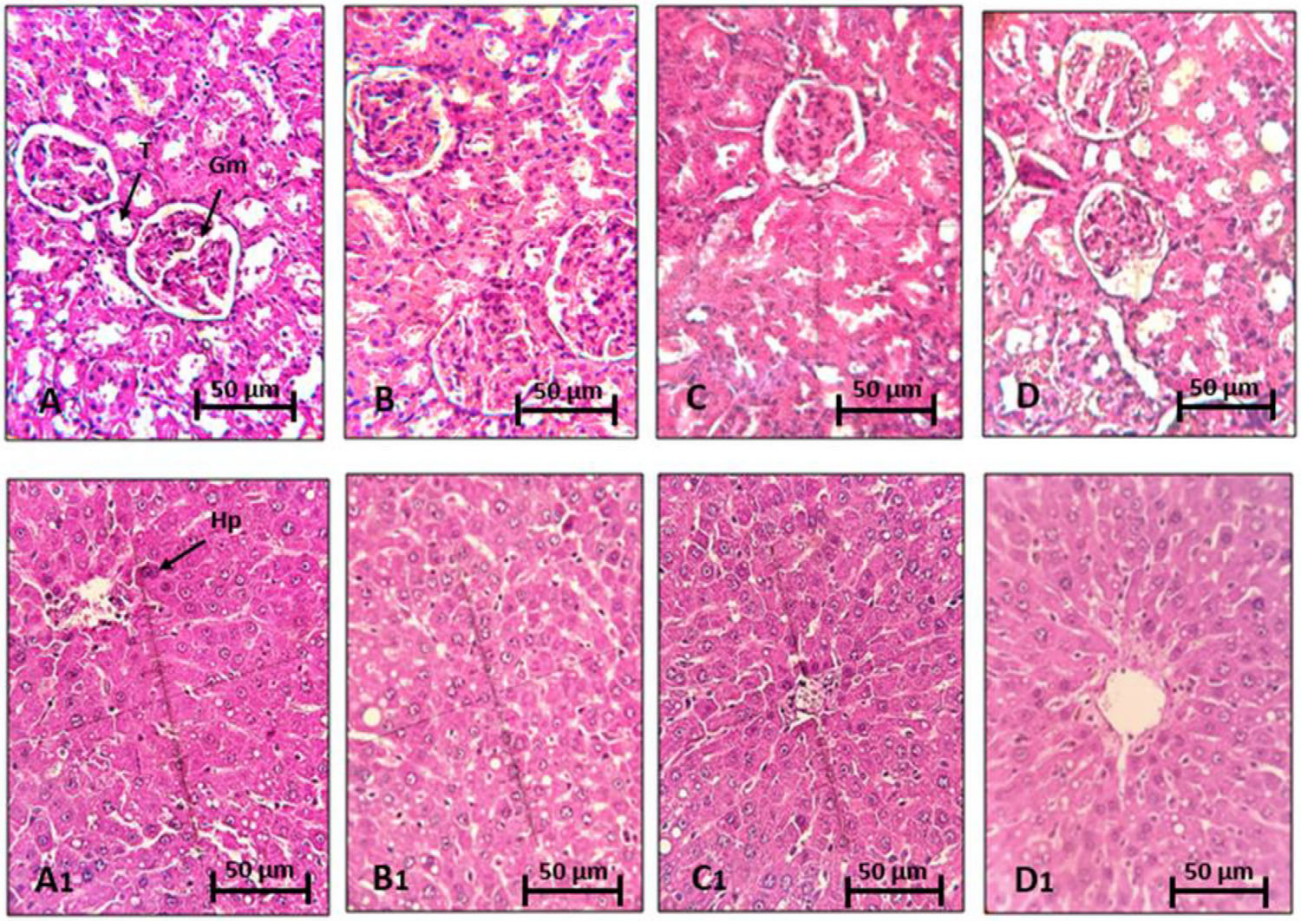
Histological analysis of the kidneys and liver of control mice (A and A1) and mice treated with ethanolic extract of *Rhus pentaphylla* leaves at doses of 100 mg/kg (B and B1), 300 mg/kg (C and C1), and 1500 mg/kg (D and D1). Gm, glomerulus; Hp, hepatocyte; T, tubule.

Histological sections of the kidneys and liver were carefully examined under a light microscope to detect any signs of toxicity or lesions. However, no alterations or abnormalities were observed at the microscopic level in these tissues. The cellular structures, tissue arrangements, and overall architecture of the kidneys and liver were intact, indicating the absence of toxicity or damage at the microscopic level. These results suggest that treatment with *R. pentaphylla* leaf extract did not cause significant histological disturbances in these organs (Figure 3).

### 2.5 Neurodevelopmental Toxicity

#### 2.5.1 Maternal Reproductive Performance and Offspring Viability

Throughout the treatment period, no deaths were observed in the females. However, it was noted that doses of 300 and 500 mg/kg of the ethanolic extract of *R. pentaphylla* leaf completely inhibited gestation in the treated females, resulting in a pregnancy index of 0%. In contrast, females treated with 100 mg/kg of the ethanolic extract exhibited a significant decrease in the pregnancy index, dropping to 33.33% compared to the control group (Table 2). Despite these effects, no toxic impacts were detected through crude visual inspection or in the body weight of the animals.

**TABLE 2:** Reproductive performance parameters of pregnant mice administered by ethanolic extract of *Rhus pentaphylla* leaf.

Parameters	Groups			
	Control	100 mg/kg	300 mg/kg	500 mg/kg
Pregnancy index (%)	100	33.33	0	0
Abortion index (%)	0	0	—	—
Deliverance index (%)	100	100	—	—

**FIGURE 4:**
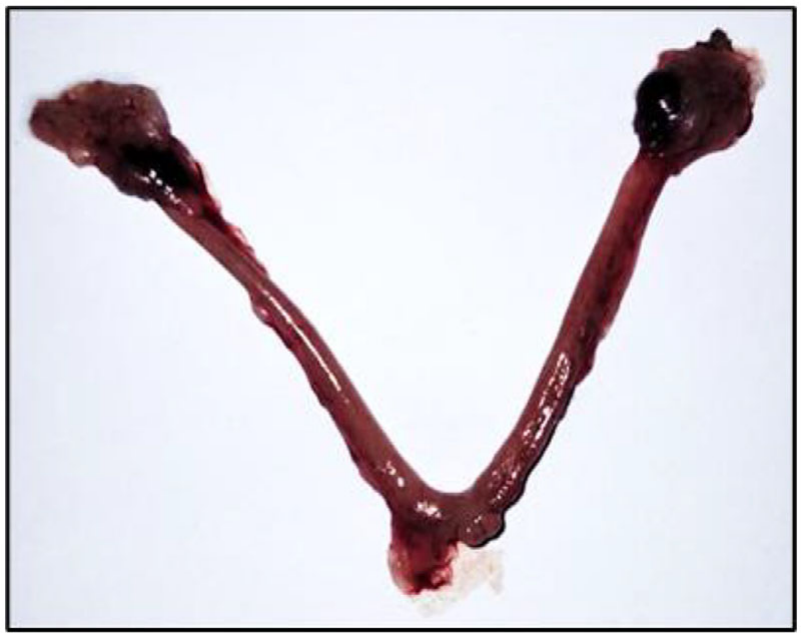
Uterus of Swiss albino mice.

At the end of the treatment, we examined the uteri of nonpregnant females and found no implants (Figure 4), indicating that treatment with the extract from *R. pentaphylla* likely inhibits implantation. When we repeated the experiment, the nonpregnant females were mated again, resulting in pregnancy and normal litters, suggesting that the effect of the extract was reversible.

Pregnant mice receiving 100 mg/kg of *R. pentaphylla* extract did not show vaginal bleeding or fetal discharge compared to the control group. In addition, there were no significant differences in body weight or pregnancy duration. The birth, viability, and lactation indices of the 100 mg/kg treated group were also similar to those of the control group. However, the significant reduction in the number of fetuses in the treated group is associated with a decreased pregnancy index of 33.33% compared to 100% in the control group. Fetal viability and litter size did not differ significantly from the control group (Table 2).

### 2.6 Offspring Neurodevelopment Evaluation

Since *R. pentaphylla* extract inhibited pregnancy at the following doses: 300 and 500 mg/kg, only control pups and pups pretreated with 100 mg/kg were subjected to evaluation for neurodevelopmental toxicity.

#### 2.6.1 Morphological Parameters

No external malformations were observed in the newborns in the pretreated groups with *R. pentaphylla* extract. In addition, prenatal exposure to the plant extract does not delay the timing of incisor eruption and the appearance of hair, eyes, and ear canal openings (Table 3).

**TABLE 3:** Effect of pretreatment with ethanolic extract of *Rhus pentaphylla* leaves on developmental parameters and viability of pups.

Parameters	Groups			
	Control	100 mg/kg	300 mg/kg	500 mg/kg
No. of pups examined	40	13	0	0
Incisor eruption	PD8 ± 0	PD6 ± 0.1	—	—
Appearance of hair	PD5 ± 0	PD6 ± 0.31	—	—
Opening of auditory canal	PD12 ± 0	PD11 ± 0.34	—	—
Eye opening	PD14 ± 0	PD13 ± 0.4	—	—
Viability index (%)	100	100	—	—
Lactation index (%)	100	96.22	—	—

*Note*: The results are presented as the mean ± SEM.

Abbreviation: PD, postnatal day.

**FIGURE 5:**
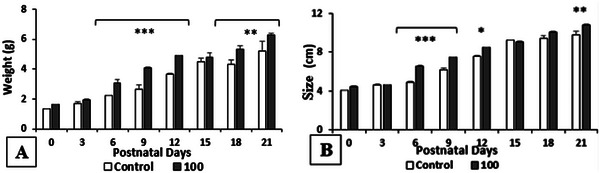
Effect of *Rhus pentaphylla* leaf on offspring's body weight (A) and size (B). The data are expressed as mean ± SEM. ***p < 0.001, **p < 0.01, *p < 0.05 versus control.

It is remarkable that the weight and size of the offspring are significantly increased by the pretreatment with *R. pentaphylla* at 100 mg/kg. Indeed, the weight is very significantly increased on days PD6, PD9, and PD12, and moderately significantly on days PD18 and PD21. In addition, the size is significantly increased by the *Rhus* extract on days PD6 and PD9, slightly significantly on day PD12, and moderately significantly on day PD21 compared to the control group (Figure 5).

### 2.7 Neurobehavioral Tests

#### 2.7.1 Surface Righting Test

**FIGURE 6:**
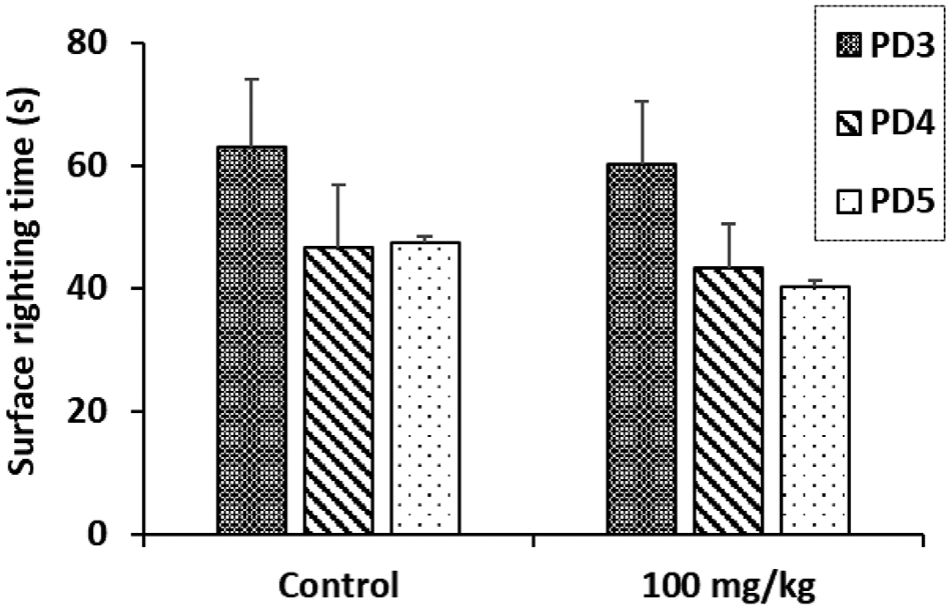
The turnaround time of pups treated prenatally with ethanolic extract of *Rhus pentaphylla* leaf in the surface righting test. Results are presented as mean ± SEM. PD, postnatal day.

The results of the surface righting test, illustrated in Figure 6, show that the extract of *R. pentaphylla* leaf at a dose of 100 mg/kg has no significant effect on the neurological integrity and motor coordination of the newborns. The time required for them to return to their normal quadruped position on the third, fourth, and fifth postnatal days (PDs) did not show any notable changes.

#### 2.7.2 Negative Geotaxis Test

**FIGURE 7:**
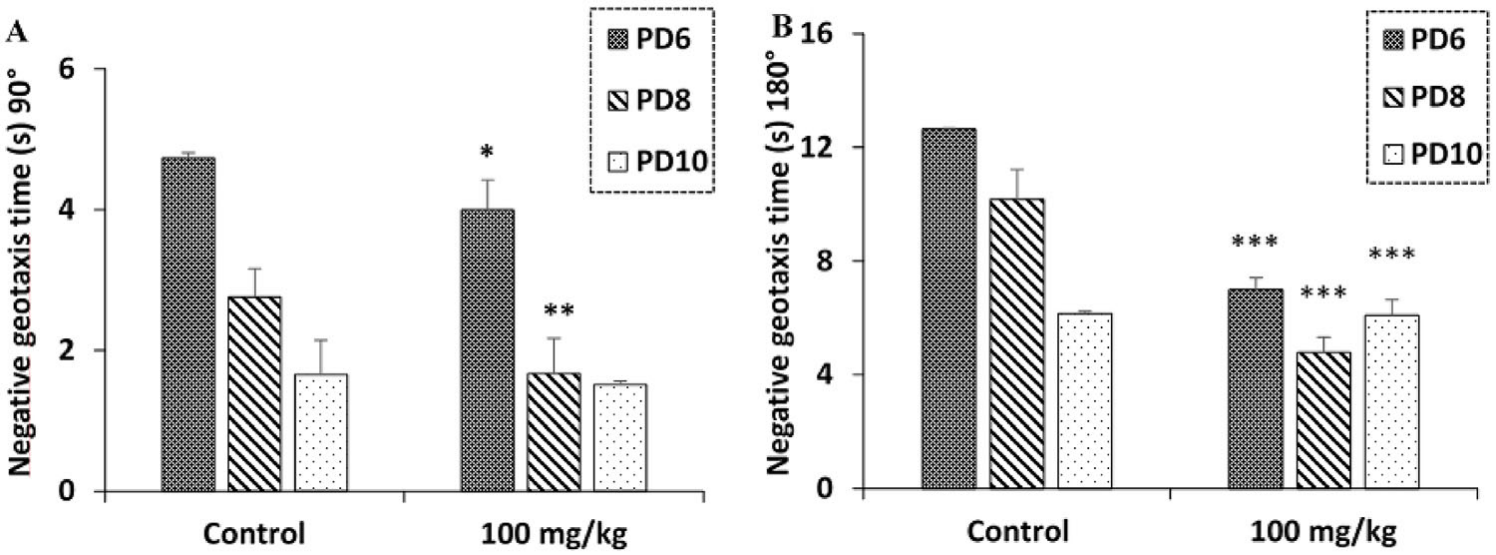
The effect of prenatal treatment with ethanolic extract of *Rhus pentaphylla* leaf on latencies to turn to 90° (A) and to turn to 180° (B) in the negative geotaxis test. Results are presented as mean ± SEM (*p < 0.05, **p < 0.01, ***p < 0.001). PD, postnatal day.

In the negative geotaxis test, one‐way ANOVA revealed that maternal exposure to *R. pentaphylla* leaf extract appears to enhance the sensorimotor performance and behavioral response to gravity in the newborns. This allowed them to turn over on an inclined surface, significantly reducing the time required to perform this reaction. This improvement was observed for both a 90° turn on the 6th day and on the 8th day and a 180° turn on the 6th, 8th, and 10th days (Figure 7).

#### 2.7.3 Rotarod Test

**FIGURE 8:**
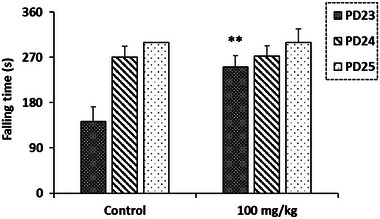
The effect of prenatal treatment with ethanolic extract of *Rhus pentaphylla* leaf on falling time in the Rotarod test. Results are presented as mean ± SEM (**p < 0.01). PD, postnatal day.

Balance and motor coordination, assessed using the Rotarod test, were significantly improved in pups exposed to *R. pentaphylla* extract compared to controls on the 23rd day. However, this improvement was not observed on the 24th and 25th PDs (Figure 8).

### 2.8 Histopathological Sections of the Brain

**FIGURE 9:**
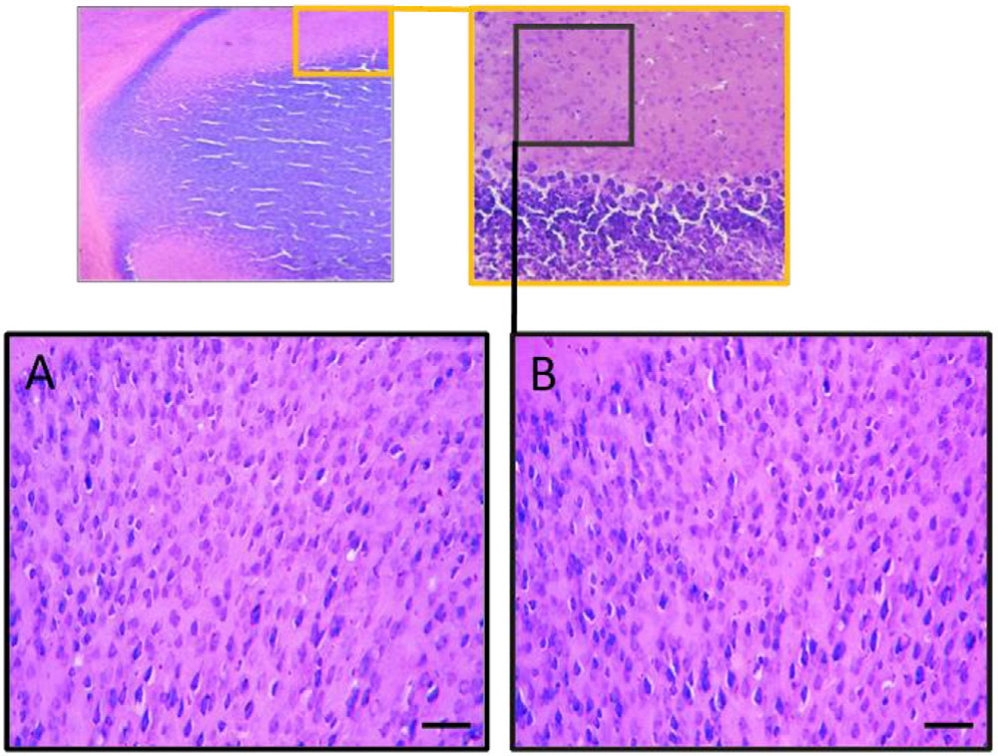
Microscopic observation of the posterior parietal cortex in the offspring pretreated with ethanolic extract of *Rhus pentaphylla* leaf after staining with cresyl violet: (A) control group, (B) group pretreated with *R. pentaphylla* leaf extract (100 mg/kg). Images were captured under a light microscope (magnification, ×200 (A, B); scale bar = 50 µm).

The study aimed to evaluate the potential toxicity of the ethanolic extract from the leaves of *R. pentaphylla* in Swiss albino mice. We monitored body weight, conducted comprehensive biochemical analyses, and performed histological examinations on treated mice to detect any adverse effects. In addition, the research extended to assessing the neuroreproductive toxicity of this extract (Figure 9).

The results indicate that the ethanolic extract of *R. pentaphylla* leaves did not cause mortality or toxicity in the mice, whether at acute or subchronic doses. The median lethal dose (LD_50_) is greater than 5 g/kg.

Our findings suggest that the ethanolic extract of *R. pentaphylla* leaves induced an increase in body weight in mice, indicating the presence of active compounds that may influence metabolism. At the administered dose of 100 mg/kg, this weight gain could reflect a pharmacological effect of the extract, possibly through stimulation of appetite, improved nutrient absorption, or metabolic modulation. Importantly, no alterations were observed in hepatic biochemical parameters (alanine aminotransferase [ALT] and aspartate aminotransferase [AST]) or liver histopathology, suggesting that the weight gain is not related to toxicity but rather to a biological activity of the plant. This hypothesis requires further investigation. Sumac, particularly *Rhus coriaria*, also possesses this capability. For example, the addition of sumac to the diet could potentially improve food intake in older adults, thereby aiding in effective malnutrition management [22]. Previous studies, such as that of Mansoub [23], have shown that the use of different levels of sumac had significant effects on feed intake and weight gain in broiler chickens. Another study revealed that the polyphenolic extract of *R. coriaria* fruits led to a significant increase in body weight and body mass index in mice [24]. This improvement was attributed to the active compounds present in sumac [25]. Among the compounds in *R. pentaphylla* that may contribute to this weight gain is caffeic acid, as confirmed by several studies. Caffeic acid has been shown to induce weight gain in diabetic animals [26]. Another study demonstrated that the intake of caffeic acid mitigated body weight loss in diabetic mice [27]. The body weight of the caffeic acid‐treated group increased throughout the experimental period in db/db mice [28]. However, no significant differences in biochemical parameters were observed between the groups exposed to *R. pentaphylla* extract and the control group.

Histological analyses of tissues from mice treated with the ethanolic extract of *R. pentaphylla* leaves revealed no significant changes in tissue architecture compared to tissues from control mice.

It should be noted that most species of *Rhus* do not exhibit toxic effects on rodents. For example, the fermented stem bark extract of *Rhus verniciflua* showed no significant toxic effects in Sprague Dawley rats after a single and repeated oral administration for 90 days [29].

Acute toxicity tests on other *Rhus* species have also yielded negative results. The hydromethanolic extract of *Rhus tripartita*, the ethanolic extract of *Rhus javanica*, and the methanolic extract of *Rhus vulgaris* showed no visible clinical signs in albino rats, rodent and non‐rodent animal models, and albino mice, respectively [30, 31, 32, 33].

Some other species of *Rhus*, such as *Rhus chinensis* (ethanolic and aqueous extracts of the fruits), *Rhus trilobata* (decoction), and *Rhus retinorrhaea* (flavonoid suspension), are generally considered nontoxic at normal concentrations. However, at high doses of 2500 or 3000 mg/kg and over extended periods, they may cause adverse effects [34, 35, 36]. Nevertheless, this is a common observation, as any substance can become toxic when consumed in excessive amounts.

Although plants are generally considered safe for long‐term use and offer health benefits, they can pose risks during pregnancy. For example, fenugreek is widely used by the population due to its numerous benefits, such as stimulating breast milk production in breastfeeding women, regulating blood sugar levels, beneficial for women with gestational diabetes [37] and its positive effects on hormonal health, including alleviating symptoms of premenstrual syndrome and menopause [38]. Fenugreek also has antioxidant and anti‐inflammatory properties [39]. However, despite these benefits, fenugreek carries risks during pregnancy, such as the possibility of spontaneous abortion and the potential for congenital malformations in mice [6, 7].

According to our observations, although the ethanolic extract of *R. pentaphylla* leaves does not exhibit toxic effects even at high doses or with long‐term use in mice, it does have an inhibitory effect on gestation at doses of 300 and 500 mg/kg, and reduces gestation at 100 mg/kg.

In addition, as mentioned in the literature, several species of *Rhus* are associated with fertility alterations in traditional uses. For instance, a decoction of *R. trilobata* leaves was traditionally consumed to promote impotence as a contraceptive measure [40]. Similarly, *R. coriaria*, when dissolved in wine, was used to tighten the entrance of the uterus due to its astringent properties [41]. Finally, *Rhus mysorensis* is reputed for its antifertility properties [42]. This reputation of *Rhus* as a contraceptive motivated us to evaluate the reproductive toxicity of *R. pentaphylla*, which had never been assessed in this context.

Although *R. pentaphylla* inhibits gestation at doses of 300 mg/kg and above, this effect is reversible. Upon repeating the experiment, the nonpregnant females were mated again, resulting in pregnancies and normal litters, suggesting that the effect of the extract was reversible.

The observed antifertility effect could be attributed to an unfavorable uterine environment for blastocyst implantation. In addition, this property may result from the chemical composition of the extract, primarily composed of phenolic compounds such as flavonoids, tannins, and phenolic acids, identified by Benamar and Bennaceur [43]. Some flavonoids have been reported to have contraceptive properties by disrupting estrogen levels [44]. Indeed, in the literature, flavonoids are known for their antifertility activity, as observed with flavonoids isolated from *Striga orobanchiodes* and *Striga lutea*, which exhibit estrogenic and antifertility activities [45]. Similarly, the methanolic extract of *Thevetia peruviana* leaves, rich in flavonoids such as kaempferol and quercetin, demonstrates antifertility properties by lowering progesterone levels in female Sprague Dawley rats [46]. Furthermore, the flavonoid extract of *Portulaca oleracea* exhibits anti‐implantation, abortive, and estrogenic properties in female albino rats [47]. Phenolic acids, such as lithospermic acid and its oxidized derivative, found in the roots of *Lithospermum ruderale*, may also exert an antigonadotropic effect [48].

Although *R. pentaphylla* has demonstrated contraceptive effects, our study revealed a significant enhancement in sensorimotor development in pups pretreated with a low dose of 100 mg/kg of *R. pentaphylla* leaf extract. Moreover, some species of *Rhus*, such as *Rhus chirindensis*, have shown the ability to reduce the severity of febrile seizures in pups by regulating plasma IL‐1 levels [49]. For example, the extract of *Rhus dentata* protected 47% of Wistar pups against seizures induced by *N*‐methyl‐d‐aspartic acid and significantly delayed the onset of seizures induced by pentylenetetrazol [50]. These findings highlight the potential of extracts from various *Rhus* species in the field of neuroprotection.

Flavonoids extracted from *R. verniciflua* could constitute effective neuroprotective agents, potentially helping to mitigate the progression of Alzheimer's disease and other neurodegenerative conditions [51]. In contrast, the glabtan isolated from *Rhus glabra* showed no effect on early postnatal physical development parameters of offspring in rats [52]. Despite these results, the exact mechanism underlying the antifertility property of *R. pentaphylla* leaf extract remains unclear.

## 3 Conclusions

In summary, this study demonstrates that the *R. pentaphylla* leaf extract does not cause overt toxicity or adverse effects on the general reproductive health of mice. In addition, it enhances the physical and neuronal development of the offspring. However, the extract exhibits contragestive properties by inhibiting gestation at certain doses, indicating its potential as a contraceptive agent. Consequently, due to its ability to interfere with pregnancy at higher doses, the use of *R. pentaphylla* should be contraindicated during pregnancy, especially at doses of 100 mg/kg or above. Nevertheless, at lower doses below 100 mg/kg, it may be possible to benefit from the plant's positive effects without the risk of contraception, although further reproductive safety evaluations are needed. Further studies are necessary to clarify the mechanisms behind these effects.

## 4 Experimental Section

### 4.1 Experimental Animals

Healthy, mature Swiss albino female and male mice (8–10 weeks old), weighing between 22 and 35 g, were randomly selected. They were provided by the central animal facility of the Faculty of Sciences‐Semlalia, Cadi Ayyad University, Marrakesh, Morocco. The animals were maintained under controlled conditions with a temperature of 22 ± 3°C, a 12‐h light/dark cycle, and free access to food and water. The mice were acclimated to the laboratory for at least 5 days before the experiment. Maximum efforts were made to minimize the number of experimental animals. All animals were treated according to accepted international standard procedures for the use of animals in laboratories, as reported in the Scientific Procedures on Living Animals Act 24.11.1986 (European Council directive: 86/609/EEC).

The euthanasia procedure complied with international standards outlined by the Organization for Economic Co‐operation and Development (OECD), the American Veterinary Medical Association (AVMA), and the European Directive 2010/63/EU. They also aligned with the ethical frameworks recommended by institutions such as the Canadian Council on Animal Care (CCAC) and the Office of Laboratory Animal Welfare (OLAW). All measures were taken to adhere to the principles of the 3Rs.

### 4.2 Plant Material

Leaves of *R. pentaphylla* were collected in April from the Sidi Rahal region, Morocco, at the geographic coordinates 31°35′05.5″ N, 7°28′11.3″ W. The botanical identification of this plant was authenticated by Professor Ahmed Ouhammou from the Laboratory of Environment and Ecology (L2E, CNRST Associated Research Unit, URAC 32), Regional Herbarium MARK, Faculty of Sciences‐Semlalia, Cadi Ayyad University, Marrakech, Morocco.

### 4.3 Preparation of the Ethanolic Extract

The leaves of *R. pentaphylla* underwent cold maceration. Initially, the leaves were dried at room temperature in the shade and then roughly powdered. Seventy grams of the powdered leaves were subjected to extraction using ethanol for 24 h with continuous stirring by a magnetic bar. The macerates were then filtered and concentrated using a rotary evaporator at 45°C, resulting in a yield of 22% ethanolic extract of *R. pentaphylla* leaves.

### 4.4 Materials and Reagents

The chemicals used in this study include ethanol, formaldehyde, and chloral hydrate, all purchased from Sigma‐Aldrich (St. Louis, MO, USA). For histological analyses, hematoxylin and eosin (H&E) as well as cresyl violet stains, both from Sigma‐Aldrich (Germany), were used for tissue section staining. Biochemical analyses were performed using a COBAS C 311 automated analyzer (Roche). Tissue sectioning was carried out with a Leica Biosystems CM1860 cryostat.

### 4.5 HPLC‐Based Analysis

The phenolic compounds in the ethanolic extract of *R. pentaphylla* leaves were separated and identified using an HPLC system. The setup included a Shimadzu (Japan) SCL‐10A pumping unit, an SIL‐10AD automated injector, and a Shimadzu UV‐Vis detector (SPD‐10A) with a wavelength range of 200–700 nm, with data collection and analysis carried out using Shimadzu software. Chromatographic separations were conducted on a reverse‐phase RP‐18 column (Agilent Technologies, 250 mm × 4.6 mm, 5.0 µm), with an Agilent RP‐18 pre‐column (10 mm × 4.6 mm), maintained at 25°C. The analysis employed two solvents in a gradient program at a flow rate of 1 mL/min, with an injection volume of 10 µL. The first solvent was a mixture of 5% acetonitrile and 95% water, while the second was a phosphate buffer (pH 2.6) in water. All solvents were of HPLC grade. Phenolic compounds were identified by comparing their retention times with those of standard reference compounds [53].

### 4.6 Acute Toxicity

For the evaluation of acute oral toxicity, 20 mice were randomly divided into 4 experimental groups (*n* = 5 per group). Three groups received a single dose of ethanolic extract of *R. pentaphylla* leaves at 1000, 2000, or 5000 mg/kg via oral gavage using a gavage syringe fitted with a metal feeding tube, appropriate for the size of the mice. The control group received distilled water by the same administration route. After treatment, the animals were monitored for 24 h to observe signs of toxicity or mortality, and the dose that killed 50% of the animals (LD_50_) was calculated [54]. Observations continued for 14 days to detect any abnormalities or delayed mortality.

### 4.7 Subchronic Toxicity

For the assessment of subchronic toxicity, 20 mice were randomly divided into four experimental groups (*n* = 5 per group). Three groups received daily oral gavage of *R. pentaphylla* leaf ethanolic extract at doses of 100, 300, or 1500 mg/kg/day for 60 days. The control group received distilled water by the same administration route. Body weight variations were recorded every 15 days during the 60‐day treatment period. The treated animals were supervised daily.

### 4.8 Biochemical Analysis

After 2 months of treatment, the mice were anesthetized and then euthanized. Blood samples were collected by cardiac puncture from all tested mice and centrifuged at 3000 RPM for 10 min to separate the plasma. The plasma was then used to measure levels of urea, creatinine, AST, ALT, and alkaline phosphatase (ALP) using commercial kits on a Roche Diagnostics Cobas C311 analyzer.

### 4.9 Histopathological Examination

The kidneys and liver were fixed in 10% formalin, then frozen and sectioned using a Cryostat Leica Biosystems CM1860. The sections, 4‐µm thick, were mounted on gelatin‐coated slides, stained with H&E, and examined under a light microscope at 400× magnification.

### 4.10 Neurodevelopmental Toxicity

#### 4.10.1 Mating Process

Adult female Swiss albino mice were housed with fertile males in cages, using one male per two females. The following morning, mating was confirmed by the presence of a vaginal plug, which was designated as Day 0 of pregnancy. Females with a vaginal plug were then housed individually and divided into four groups of five each for treatment.

#### 4.10.2 Gestation Period

Pregnant control mice received distilled water. The other three groups were treated with the ethanolic extract of *R. pentaphylla* leaf orally at increasing doses of 100, 300, and 500 mg/kg daily throughout the all‐gestational period. The females were monitored throughout the experimental period, with daily body weight measurements taken from the day the vaginal plug appeared until parturition. Their condition was observed to detect any signs of toxicity or effects such as premature birth, abortion, morbidity, or mortality. During the gestation period, two indices were calculated: the pregnancy index (the percentage of pregnant females showing a vaginal plug) and the abortion index (the percentage of spontaneously aborted offspring).

On Day 21, in nulliparous females, a laparotomy was performed, and the uterine horns were exteriorized and extended to check for the presence of dead fetuses.

#### 4.10.3 Second Reproduction Phase

We repeated the same experiment conducted previously, but instead of sacrificing the nonpregnant females on the 21st day, we initiated a new reproduction phase. At the end of the treatment period, the nonpregnant females were reintroduced into cages with males for another reproduction trial.

#### 4.10.4 Offspring Development

The progeny of females who gave birth were examined for noticeable physical malformations. Body weight and length were measured on PD 0, 3, 6, 9, 12, 15, 18, and 21. Developmental milestones such as hair appearance, incisor eruption, and the opening of eyes and ears were recorded. Several indices were calculated: the delivery index (percentage of females giving birth among pregnant mice), the viability index (live litters on the fourth day of lactation as a percentage of live litters), and the lactation index (number of live offspring on Day 21 as a percentage of the number of live offspring). In total, 40 offspring were obtained from the control group, composed of 5 females, whereas only 13 offspring were recorded in the group pretreated with 100 mg/kg of *R. pentaphylla* extract, also composed of 5 females.

### 4.11 Offspring Neurodevelopment Tests

#### 4.11.1 Surface Righting Test

This experiment was carried out on PND 3, 4, and 5. Each neonate was placed on its back on a smooth horizontal surface, and the time taken for all four limbs to make contact with the surface was measured. The duration of this test was 2 min in total [16].

#### 4.11.2 Negative Geotaxis Test

We conducted the experiment on PND 6, 8, and 10. The pups were positioned head down on a 45° inclined plane, and the time taken to turn 90° and 180° with their head up was recorded. Each animal was observed for a maximum of 2 min [55].

#### 4.11.3 Cliff Avoidance Test

We performed experiments on PND 6, 8, and 10. The offspring were placed on a platform elevated 10 cm above a table, with their forepaws and muzzle positioned at the edge. The time taken for them to turn back to avoid falling was recorded, with a maximum observation period of 2 min [16].

#### 4.11.4 Rotarod Test

On PND 23, 24, and 25, the pups were placed on a rotarod device with a diameter of 6 cm, rotating at 20 RPM. This test assessed the motor skills and coordination of the neonates by measuring the duration they could remain on the rotating apparatus without falling. The test lasted for 5 min [56].

#### 4.11.5 Histopathological Sections

Histopathological studies were executed to examine the effects of the ethanol extract of *R. pentaphylla* leaf on brain function. The pups were anesthetized and perfused intracardially with 0.9% physiological saline, followed by 10% formalin. The brains were then removed, fixed in 10% formalin, frozen, and sectioned using a Cryostat Leica Biosystems CM1860. Sections (40 µm) were mounted on gelatin‐coated slides, stained with Cresyl violet, and analyzed under a light microscope. The overall histological appearance of the brain was assessed.

### 4.12 Statistical Analysis

The data were analyzed using Sigma Plot for Windows, version 12.5. Results are presented as means ± SEM. Statistical analysis was performed using one‐way ANOVA followed by Tukey's post hoc test. *p* < 0.05 was considered statistically significant.

## Author Contributions

**Fatimazahra Agouram**: conceptualization, data curation, formal analysis, investigation, methodology, visualization, writing – original draft, writing – review and editing, resources. **Aicha Ezoubeiri**: methodology. **Abderrahman Chait**: conceptualization, investigation, supervision, validation, resources. **Stefania Garzoli**: validation, writing – review and editing, supervision. **Zahra Sokar**: conceptualization, investigation, supervision, validation, writing – review and editing, resources.

## Ethics Statement

All procedures were rigorously carried out in full compliance with internationally recognized ethical standards, particularly those outlined in European Directive 2010/63/EU.

## Conflicts of Interest

The authors declare no conflicts of interest.

## Data Availability

The data that support the findings of this study are available from the corresponding author upon reasonable request.

## References

[1] K. Karunamoorthi , K. Jegajeevanram , J. Vijayalakshmi , and E. Mengistie , “Traditional Medicinal Plants,” Journal of Evidence‐Based Complementary and Alternative Medicine 18, no. 1 (2013): 67–74, 10.1177/2156587212460241.

[2] M. Elachouri , “Ethnobotany/Ethnopharmacology, and Bioprospecting: Issues on Knowledge and Uses of Medicinal Plants by Moroccan People,” Natural Products and Drug Discovery, ed. S. C. Mandal , V. Mandal , and T. Konishi (Elsevier, 2018): 105–118, 10.1016/B978-0-08-102081-4.00005-8.

[3] B. Guldiken , G. Ozkan , G. Catalkaya , F. D. Ceylan , I. Ekin Yalcinkaya , and E. Capanoglu , “Phytochemicals of Herbs and Spices: Health Versus Toxicological Effects,” Food and Chemical Toxicology 119 (2018): 37–49, 10.1016/j.fct.2018.05.050. 29802945

[4] S. M. Illamola , O. U. Amaeze , L. V. Krepkova , et al., “Use of Herbal Medicine by Pregnant Women: What Physicians Need to Know,” Frontiers in Pharmacology 10 (2020): 1483, 10.3389/fphar.2019.01483. 31998122 PMC6962104

[5] N. Bernstein , M. Akram , Z. Yaniv‐Bachrach , and M. Daniyal , “Is It Safe to Consume Traditional Medicinal Plants During Pregnancy?,” Phytotherapy Research 35, no. 4 (2021): 1908–1924, 10.1002/ptr.6935. 33164294

[6] L. Khalki , S. B. M'Hamed , M. Bennis , A. Chait , and Z. Sokar , “Evaluation of the Developmental Toxicity of the Aqueous Extract From *Trigonella foenum‐graecum* (L.) in Mice,” Journal of Ethnopharmacology 131, no. 2 (2010): 321–325, 10.1016/j.jep.2010.06.033. 20600755

[7] S. Oufquir , M. Ait Laaradia , Z. El Gabbas , et al., “ *Trigonella foenum‐graecum* L. Sprouted Seed Extract: Its Chemical HPLC Analysis, Abortive Effect, and Neurodevelopmental Toxicity on Mice,” Evidence‐Based Complementary and Alternative Medicine 2020 (2020): 1615794, 10.1155/2020/1615794. 32328121 PMC7166263

[8] A. Demmers , J. J. Mes , R. G. Elbers , and R. H. Pieters , “Possible Harms of *Momordica charantia* L. in Humans; a Systematic Review,” preprint, Medrxiv, October 22, 2022, 10.1101/2022.10.22.22281390.

[9] I. R. Kashani , M. Ansari , K. Mehrannia , K. Moazzemi , and S. V. Joybary , “Teratogenic Effects of *Origanum vulgare* Extract in Mice Fetals,” Tehran University Medical Journal 71, no. 8 (2013): 502–508, http://tumj.tums.ac.ir/article‐1‐5606‐en.html.

[10] M. R. Souza , E. C. B. Brito , L. S. Furtado , et al., “Maternal‐Fetal Toxicity of *Strychnos pseudoquina* Extract Treatment During Pregnancy,” Journal of Ethnopharmacology 311 (2023): 116459, 10.1016/j.jep.2023.116459. 37023837

[11] G. E. Kisby , H. Moore , and P. S. Spencer , “Animal Models of Brain Maldevelopment Induced by Cycad Plant Genotoxins,” Birth Defects Research Part C: Embryo Today: Reviews 99, no. 4 (2013): 247–255, 10.1002/bdrc.21052. 24339036 PMC4183057

[12] D. L. Dantas , M. da C. Alves , G. M. S. Dantas , et al., “Supplementation With *Moringa oleifera* Lam Leaf and Seed Flour During the Pregnancy and Lactation Period of Wistar Rats: Maternal Evaluation of Initial and Adult Neurobehavioral Development of the Rat Progeny,” Journal of Ethnopharmacology 325 (2024): 117904, 10.1016/j.jep.2024.117904. 38342151

[13] M. F. F. T. de Melo , D. E. Pereira , R. L. Moura , et al., “Maternal Supplementation With Avocado (*Persea americana* Mill.) Pulp and Oil Alters Reflex Maturation, Physical Development, and Offspring Memory in Rats,” Frontiers in Neuroscience 13 (2019): 9, 10.3389/fnins.2019.00009. 30728763 PMC6351466

[14] G. M. Abu‐Taweel , “Cardamom (*Elettaria cardamomum*) Perinatal Exposure Effects on the Development, Behavior and Biochemical Parameters in Mice Offspring,” Saudi Journal of Biological Sciences 25, no. 1 (2018): 186–193, 10.1016/j.sjbs.2017.08.012. 29379379 PMC5775110

[15] G. Bolumbu and K. V. Mitha , “ *Centella asiatica* and Protection in Neurodevelopment,” Treatments, Nutraceuticals, Supplements, and Herbal Medicine in Neurological Disorders, ed. C. R. Martin , V. B. Patel , and V. R. Preedy (Academic Press, 2023), 891–908, 10.1016/b978-0-323-90052-2.00042-1.

[16] S. K. Lee , H. S. Jung , W. K. Eo , S. Y. Lee , S. H. Kim , and B. S. Shim , “ *Rhus verniciflua* Stokes Extract as a Potential Option for Treatment of Metastatic Renal Cell Carcinoma: Report of Two Cases,” Annals of Oncology 21 (2010): 1383–1385, 10.1093/annonc/mdq154. 20363807

[17] F. Agouram , Z. Sokar , and A. Chait , “Evaluation of Antinociceptive Activity, Antioxidant Properties and Total Phenolic Content of the Ethanolic Extracts of *Rhus pentaphylla* Leaves and Fruits From Morocco,” Journal of Animal & Plant Sciences 33, no. 6 (2023): 1304–1313, 10.36899/JAPS.2023.6.0670.

[18] C. Itidel , M. Chokri , B. Mohamed , and Z. Yosr , “Antioxidant Activity, Total Phenolic and Flavonoid Content Variation Among Tunisian Natural Populations of *Rhus tripartita* (Ucria) Grande and *Rhus pentaphylla* Desf,” Industrial Crops and Products 51 (2013): 171–177, 10.1016/j.indcrop.2013.09.002.

[19] A. Djihad , “Étude bibliographique sur la phytochimie de quelques espèces du genre Rhus” (PhD diss., Université Kasdi Merbah, 2013), https://dspace.univ‐ouargla.dz/jspui/handle/123456789/8087.

[20] H. Fadhil , F. Mraihi , J. K. Cherif , and M. Sökmen , “Comparative Study on Total Polyphenols Content of Tunisian Wild *Rhus pentaphylla* Fruit Extracts and the Evaluation of Their Biological Activities,” Italian Journal of Food Science 31, no. 2 (2019): 224–232.

[21] H. Ghouila , W. Haddar , M. B. Ticha , et al., “ *Rhus pentaphylla* Bark as a New Source of Natural Colorant for Wool and Silk Fibers,” Journal of the Tunisian Chemical Society 16 (2014): 95–102.

[22] N. Soleymani Majd , S. Coe , H. Lightowler , and P. S. Thondre , “The Effect of High‐Polyphenol Sumac (*Rhus coriaria*) on Food Intake Using Sensory and Appetite Analysis in Younger and Older Adults: A Randomized Controlled Trial,” Food Science & Nutrition 11, no. 7 (2023): 3833–3843, 10.1002/fsn3.3369. 37457172 PMC10345699

[23] N. H. Mansoub , “Effect of Different Levels of Sumac Powder (*Rhus coriaria* L.) on Performance, Carcass, and Blood Parameters of Broiler Chickens,” Annals of Biological Research 2, no. 5 (2011): 647–652.

[24] N. F. Al‐Hamdany and K. A. Al‐Flayeh , “The Effects of Polyphenol Extract of Sumac (*Rhus coriaria*) Fruits on Body Weights, Lipid Profile and Leptin Hormon Levels in Experimental Mice,” Rafidain Journal of Science 28, no. 4 (2019): 61–75, 10.33899/rjs.2019.163302.

[25] G. E. S. Batiha , O. M. Ogunyemi , H. M. Shaheen , et al., “ *Rhus coriaria* L.(Sumac), a Versatile and Resourceful Food Spice With Cornucopia of Polyphenols,” Molecules 27, no. 16 (2022): 5179. 36014419 10.3390/molecules27165179 PMC9414570

[26] N. Oršolić , D. Sirovina , D. Odeh , et al., “Efficacy of Caffeic Acid on Diabetes and Its Complications in the Mouse,” Molecules 26, no. 11 (2021): 3262, 10.3390/molecules26113262. 34071554 PMC8199327

[27] R. G. Matowane . “Synthesis, Characterisation and Evaluation of the Antidiabetic and Antioxidative Properties of Caffeic Acid and Ferulic Acid‐Zinc (II) Complexes” (PhD diss., Central University of Technology, 2022), http://hdl.handle.net/11462/2554.

[28] G. R. Matowane , S. S. Mashele , T. J. Makhafola , and C. I. Chukwuma , “The Ameliorative Effect of Zinc Acetate With Caffeic Acid in the Animal Model of Type 2 Diabetes,” Biomedicine & Pharmacotherapy 163 (2023): 114779, 10.1016/j.biopha.2023.114779. 37119739

[29] S.‐H. Shin , K.‐H. Koo , J.‐S. Bae , et al., “Single and 90‐Day Repeated Oral Dose Toxicity Studies of Fermented *Rhus verniciflua* Stem Bark Extract in Sprague–Dawley Rats,” Food and Chemical Toxicology 55 (2013): 617–626, 10.1016/j.fct.2013.01.043. 23416650

[30] L. Sudheshna , C. L. Sukesh Krishna , and A. Srinivasa Rao , “Anti Urolithiatic Activity of *Rhus mysorensis* Against Experimentally Induced Urolithiasis in Male Albino Rats,” Journal of Medical Sciences and Clinical Research 3 (2015): 7546–7551, 10.18535/jmscr/v3i9.40.

[31] Z. B. Barka , C. Aouadhi , M. Tlili , et al., “Evaluation of the Anti‐Diarrheal Activity of the Hydromethanolic Root Extract of *Rhus tripartita* (Ucria) (Anacardiaceae),” Biomedicine & Pharmacotherapy 83 (2016): 827–834, 10.1016/j.biopha.2016.07.055. 27501500

[32] J.‐H. Park , J.‐S. Ra , J. E. Kwon , et al., “Evaluation of Genetic Toxicity, Acute and Sub‐Chronic Oral Toxicity and Systemic Safety of *Agrimonia pilosa* and *Rhus gall* 50% Ethanolic Extract Mixture (APRG64) In Vitro and In Vivo (Rodent and Non‐Rodent Animal Models),” Toxicological Research 36 (2020): 367–406, 10.1007/s43188-020-00042-5. 33005596 PMC7494697

[33] A. Mutuku , L. Mwamburi , L. Keter , et al., “Evaluation of the Antimicrobial Activity and Safety of *Rhus vulgaris* (Anacardiaceae) Extracts,” BMC Complementary Medicine and Therapies 20, no. 1 (2020): 272, 10.1186/s12906-020-03063-7. 32912200 PMC7488075

[34] Z. Wu , Y. Ma , L. Zhao , S. Cai , and G. Cheng , “Acute and Subchronic Toxicities of the Ethanol and Hot‐Water Extracts From Chinese Sumac (*Rhus chinensis* Mill.) Fruits by Oral Administration in Rats,” Food and Chemical Toxicology 119 (2018): 14–23, 10.1016/j.fct.2018.06.009. 29886233

[35] L. Varela‐Rodríguez , B. Sánchez‐Ramírez , I. S. Rodríguez‐Reyna , et al., “Biological and Toxicological Evaluation of *Rhus trilobata* Nutt. (*Anacardiaceae*) Used Traditionally in Mexico Against Cancer,” BMC Complementary Medicine and Therapies 19, no. 1 (2019): 153, 10.1186/s12906-019-2566-9. PMC6604276 31262287

[36] S. Alqasoumi , A. Galal , A. Gamal , M. Al‐Yahya , and S. Rafatullah , “Antinociceptive, Anti‐Inflammatory, and Antipyretic Effects of a Flavonoidal Mixture From the Leaf Surface of *Rhus retinorrhaea* ,” Farmacia 57, no. 3 (2009): 346–354, https://www.researchgate.net/publication/239610797.

[37] A. Srivastava , Z. Singh , V. Verma , and T. Choedon , “Potential Health Benefits of Fenugreek With Multiple Pharmacological Properties,” Research Anthology on Recent Advancements in Ethnopharmacology and Nutraceuticals (IGI Global, 2022), 672–687, 10.4018/978-1-6684-3546-5.ch034.

[38] E. Akhtari , M. Ram , S. M. A. Zaidi , A. M. Marques , R. Rahimi , and R. Bahramsoltani , “Fenugreek (*Trigonella foenum‐graecum* L.) in Women's Health: A Review of Clinical Evidence, Pharmacological Mechanisms, and Traditional Use,” Journal of Herbal Medicine 43 (2023): 100816, 10.1016/j.hermed.2023.100816.

[39] Y. Liu , R. Kakani , and M. G. Nair , “Compounds in Functional Food Fenugreek Spice Exhibit Anti‐Inflammatory and Antioxidant Activities,” Food Chemistry 131, no. 4 (2012): 1187–1192, 10.1016/j.foodchem.2011.09.102.

[40] D. Kumar , A. Kumar , and O. Prakash , “Potential Antifertility Agents From Plants: A Comprehensive Review,” Journal of Ethnopharmacology 140, no. 1 (2012): 1–32, 10.1016/j.jep.2011.12.039. 22245754

[41] J. T. Noonan Jr and J. T. Noonan , Contraception: A History of Its Treatment by the Catholic Theologians and Canonists (Harvard University Press, 2012), https://www.amazon.com/dp/0674168526.

[42] G. Azam , S. G. Jayanna , A. Nelliankla , et al., “Evaluation of In Vitro Antioxidant, Anti‐Inflammatory, Anticoagulant and Antiplatelet Potential of *Rhus mysorensis* ,” Biomedicine 41, no. 4 (2021): 724–731, 10.51248/.v41i4.1365.

[43] H. Benamar and M. Bennaceur , “Bioactive Compound Contents and Biological Activities of the Algerian Medicinal Plant *Rhus pentaphylla* (Jacq.) Desf. (Anacardiaceae),” Biology and Life Sciences Forum 6, no. 1 (2021): 68, 10.3390/Foods2021-10915.

[44] M. Abdi , H. Karimzadeh , A. Jourabchi , A. Khameneh , and A. Abedelahi , “Anti‐Infertility Roles of Flavonoids: Insights Into the Female Reproductive System,” Molecular Biology Reports 52, no. 1 (2025): 495, 10.1007/s11033-025-10579-z. 40404937

[45] N. Vasudeva and S. K. Sharma , “Post‐Coital Antifertility Activity of *Hibiscus rosa‐sinensis* Linn. Roots,” Evidence‐Based Complementary and Alternative Medicine 5, no. 1 (2008): 91–94, 10.1093/ecam/nem003. 18317554 PMC2249740

[46] J. Samanta , S. Bhattacharya , and A. C. Rana , “Antifertility Activity of *Thevetia peruviana* (Pers.) K. Schum Leaf in Female Sprague‐Dawley Rat,” Indian Journal of Pharmacology 48, no. 6 (2016): 669, 10.4103/0253-7613.194861. 28066105 PMC5155468

[47] H. Nayaka , R. L. Londonkar , and M. K. Umesh , “Evaluation of Potential Antifertility Activity of Total Flavonoids, Isolated From *Portulaca oleracea* L. on Female Albino Rats,” International Journal of PharmTech Research 6 (2014): 783–793.

[48] W. L. Applequist , M. C. Bridges , and D. E. Moerman , “North American Fertility–Regulating Botanicals: A Review,” Economic Botany 76 (2022): 84–113, 10.1007/s12231-021-09532-5.

[49] L. Qulu , W. M. Daniels , V. Russell , and M. V. Mabandla , “ *Searsia chirindensis* Reverses the Potentiating Effect of Prenatal Stress on the Development of Febrile Seizures and Decreased Plasma Interleukin‐1β Levels,” Neuroscience Research 103 (2016): 54–58, 10.1016/j.neures.2015.08.004. 26320878

[50] M. E. Pedersen , R. A. Baldwin , J. Niquet , et al., “Anticonvulsant Effects of *Searsia dentata* (Anacardiaceae) Leaf Extract in Rats,” Phytotherapy Research 24, no. 6 (2010): 924–927, 10.1002/ptr.3016. 19953526

[51] N. Cho , J. H. Choi , H. Yang , et al., “Neuroprotective and Anti‐Inflammatory Effects of Flavonoids Isolated From *Rhus verniciflua* in Neuronal HT22 and Microglial BV2 Cell Lines,” Food and Chemical Toxicology 50, no. 6 (2012): 1940–1945, 10.1016/j.fct.2012.03.052. 22465834

[52] Г. Г. Рахмонова , Н. Г. Абдулладжанова , Р. Н. Рахимов , К. Р. У. Баратов , and Р. А. Якубова , “Изучение Репродуктивной Токсичности Суммы Полифенолов Из Растения *Rhus glabra* На Лабораторных Крысах,” Universum: Химия и Биология 6, no. 96 (2022): 63–70, 10.32743/UniChem.2022.96.6.13633.

[53] F. Z. Marhoume , M. A. Laaradia , Y. Zaid , et al., “Anti‐Aggregant Effect of Butanolic Extract of *Rubia tinctorum* L on Platelets In Vitro and Ex Vivo,” Journal of Ethnopharmacology 241 (2019): 111971, 10.1016/j.jep.2019.111971. 31153862

[54] A. Khattabi , N. Rhalem , A. Chabat , S. Skalli , and P. R. Soulaymani‐Bencheich , Revue de toxicologie Maroc, N°5 –2ème trimestre (Publication officielle du Centre Anti Poison du Maroc Ministère de la santé, 2010), https://www.academia.edu/26257840/.

[55] A. R. Mesquita , J. M. Pêgo , T. Summavielle , P. Maciel , O. F. X. Almeida , and N. Sousa , “Neurodevelopment Milestone Abnormalities in Rats Exposed to Stress in Early Life,” Neuroscience 147, no. 4 (2007): 1022–1033, 10.1016/j.neuroscience.2007.04.007. 17587501

[56] H. Shiotsuki , K. Yoshimi , Y. Shimo , et al., “A Rotarod Test for Evaluation of Motor Skill Learning,” Journal of Neuroscience Methods 189, no. 2 (2010): 180–185, 10.1016/j.jneumeth.2010.03.026. 20359499

